# An Integrated Approach to Identifying International Foodborne Norovirus Outbreaks^1^

**DOI:** 10.3201/eid1703.100979

**Published:** 2011-03

**Authors:** Linda Verhoef, Roger D. Kouyos, Harry Vennema, Annelies Kroneman, Joukje Siebenga, Wilfrid van Pelt, Marion Koopmans, on behalf of the Foodborne Viruses in Europe Network

**Affiliations:** Author affiliations: National Institute for Public Health and the Environment (RIVM), Bilthoven, the Netherlands (L. Verhoef, H. Vennema, A. Kroneman, J. Siebenga, W. van Pelt, M. Koopmans);; Swiss Federal Institution of Technology (ETH) Zurich, Switzerland (R. Kouyos)

**Keywords:** Viruses, norovirus, foodborne infections, epidemiology, surveillance, research

## Abstract

International foodborne norovirus outbreaks can be difficult to recognize when using standard outbreak investigation methods. In a novel approach, we provide step-wise selection criteria to identify clusters of outbreaks that may involve an internationally distributed common foodborne source. After computerized linking of epidemiologic data to aligned sequences, we retrospectively identified 100 individually reported outbreaks that potentially represented 14 international common source events in Europe during 1999–2008. Analysis of capsid sequences of outbreak strains (n = 1,456), showed that ≈7% of outbreaks reported to the Foodborne Viruses in Europe database were part of an international event (range 2%–9%), compared with 0.4% identified through standard epidemiologic investigations. Our findings point to a critical gap in surveillance and suggest that international collaboration could have increased the number of recognized international foodborne outbreaks. Real-time exchange of combined epidemiologic and molecular data is needed to validate our findings through timely trace-backs of clustered outbreaks.

## Abstract 2

Surveillance gaps can be bridged through analysis of combined molecular and epidemiologic data.

Noroviruses are the most prevalent causative agents of acute viral gastroenteritis in the community (1–4). Currently, 5 norovirus genogroups have been described and subdivided into at least 40 genotypes (5,6), but in recent years, most clinical effects have been caused by viruses from a single genotype in genogroup II, GII.4 (7–10). The symptoms of norovirus disease are usually mild and self-limiting, but there is some evidence the disease can contribute to proportion of deaths (11,12). Infection occurs by way of the gastrointestinal tract after contact with infected persons, after ingestion of contaminated food or aerosols, or through environmental contamination (13,14).

Because the different modes of transmission call for quite distinct control measures, it is important to assess which proportion of disease can be attributed to which mode of transmission. However, this question is difficult to answer. Due to the high rate and rapidity of secondary spread of norovirus infection following a foodborne introduction, outbreaks initially linked to a food source may appear to be person-to-person (PTP) outbreaks by the time they are recognized. Even if a foodborne source is suspected, confirmation of the source is complicated. Virus detection in food commodities is possible but hampered by such factors as low levels of norovirus in food, food matrix complexity, genetic variability of norovirus (15), the absence of an efficient cell culture system to propagate human noroviruses (16), and the unavailability of leftover food for pathogen detection.

Given the globalization of the food market, diffuse international outbreaks are likely (17,18). For public health officials, these may seem to be regular PTP outbreaks because infection of 1 or a few persons with viruses through food consumption will go unnoticed unless secondary spread occurs or the contaminated food is consumed by multiple persons, which may trigger an investigation to identify a source. However, identification of international links is complicated. Viruses remain infectious in frozen ready-to-eat products over prolonged periods, and linked outbreaks are likely to be separated in time (19). Other problems are virus mutation rate, which results in nonidentical strains from a common source (20); sewage contamination with multiple nonsimilar strains during production of shellfish or crops (21); underreporting of cases (22,23); and incompleteness of outbreak reports (24,25). Other complicating factors include the unknown background level of viruses in foods, the environment, or asymptomatic shedders. Clearly, methods combining molecular and basic epidemiologic criteria are needed to assist public health efforts to identify international foodborne outbreaks.

For this reason, we performed a retrospective analysis of norovirus outbreak surveillance data collected since 1999 by Food-Borne Viruses in Europe (FBVE), a combined laboratory and epidemiology network (6). Although the name FBVE suggests a foodborne focus, the network actually investigates outbreaks of viral gastroenteritis with all modes of transmission. It seeks to obtain a comprehensive overview of viral activity in the community and to enable capture of foodborne norovirus outbreaks that have evaded recognition. Strain sequences from outbreaks linked to a common source are expected to be more similar than strains from outbreaks with a different source (26). We sought to quantify strain variability within and among molecular sequence clusters of multiple outbreaks to identify outbreaks with probable links to other outbreaks. Our goal was to retrospectively identify potential common-source events not detected by routine investigations and also to provide criteria that may assist in detecting such events.

## Methods

### Definitions

A norovirus outbreak was reported to FBVE when it included a minimum of 2 patients in the same area within 2 days who had >2 instances of vomiting and/or watery diarrhea within a 24-hour period (6,27). A gastroenteritis outbreak was ascribed to norovirus on the basis of compatible descriptive epidemiology and laboratory confirmation in at least 2 of 5 feces samples tested (28). An outbreak strain was defined as a sequenced norovirus strain considered representative of an outbreak (found preferably in >2 samples from patients in the same outbreak). If dissimilar sequences were detected, multiple strains were considered representative. A genotype is a group of closely related noroviruses, i.e., showing >80% similarity in the complete capsid amino acid sequence. Genotypes can be assigned based on shorter sequences if a full capsid was previously identified and sequenced for comparison (29). In this report, a cluster refers to a molecular cluster of multiple outbreaks, not an epidemiologic cluster of patients in 1 outbreak. A cluster of similar sequences is a group of outbreak strain sequences in the same genotype that show a minimal number of mutations within the region of overlap; the exact number of mutations depends on the sequence length in the region and the cutoff value used to define similarity. A cluster of identical sequences is a group of outbreak strain sequences with the highest possible similarity (100%). According to reporting standards of the FBVE network (24,30), the suspected mode of transmission during an outbreak was considered foodborne when the infection was related to consumption of food contaminated during its production or processing; food handler–borne (FHB) when infection related to food prepared by an infected food-handler; person-borne when it related to direct contact with infected persons; and unknown (UN) when no mode could be identified.

### Selection of Strains Representing Outbreaks

From January 1999 through November 2008, the FBVE network collected molecular information on a total of 5,499 norovirus outbreaks in Denmark, Finland, France, England and Wales, Germany, Hungary, Ireland, Italy, the Netherlands, Norway, Slovenia, Spain, and Sweden (24,30). Strengths and limitations of the FBVE data collection have been described (24). FBVE data are reported aggregated at outbreak level. Consequently, throughout the analysis here described, a strain represents an outbreak (i.e., outbreak-representative strain), and a cluster is a molecular cluster of outbreaks (i.e., cluster of outbreak-representative strains).

Because the norovirus genome shows its highest variability in the capsid, comparing sequences from this region will yield the lowest number of identical strains (31). Therefore, regions C and D, both located in open reading frame (ORF) 2 at the capsid gene, were our regions of choice for identification of linked outbreaks. All norovirus outbreak strains reported to the FBVE network from January 1999 through November 2008 were included if a full or partial capsid sequence was involved. This yielded 1,504 outbreak-representative strain sequences reported by all above-mentioned countries. Sequence lengths varied between 90 nt and 1,640 nt. We used sequences including ORF2 nt positions 1–300 (93%) and other targets within ORF2 nt positions 300–1,620 (7%), including full capsid genes (8%).

### Assignment of Genotypes

We classified genotypes on the basis of their similarity to reference strains representing known genotypes using the norovirus typing tool (www.rivm.nl/mpf/norovirus/typingtool). In this study, the ORF2 reference set was used for genotyping.

### Alignments of Strains Representing Outbreaks

Nucleotide sequence alignment and similarity calculation according to the neighbor-joining method were performed for all ORF2 outbreak strain sequences within genotypes by using Bionumerics 5.1 (Applied Maths, Kortrijk, Belgium). If sequences from non-overlapping nucleotide positions were included, these sequences were separately aligned within the involved nucleotide positions.

### Analysis of Clustering Strains Representing Outbreaks

Alignments were imported into the R project version 2.8.0 (http://cran.r-project.org) for analysis in 6 steps (Figure). In step 1, the APE (32) and the seqinR (33) packages in R were used to assign numbers to clusters of identical outbreak strain sequences, according to pair-wise comparison of strains within genotypes. Cluster numbers were assigned to enable rapid and computerized linking of the large molecular and epidemiologic datasets and allow systematic statistical analysis of combined data.

**Figure:**
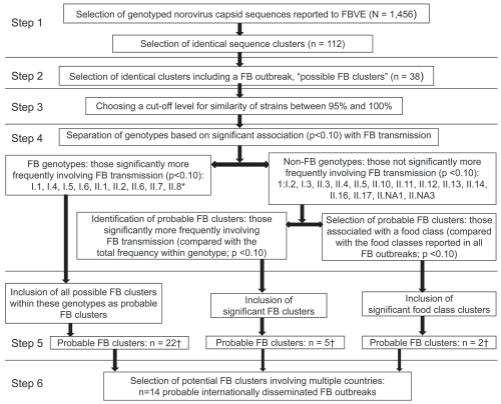
Selection of foodborne (FB) clusters of strains potentially representing internationally disseminated common-source outbreaks. Selection involved 6 steps, according to combined epidemiologic and molecular criteria. Each analyzed strain represented an outbreak. *See Table A1; †see Table A2.

In step 2, the characteristics of outbreak strain sequence clusters were compared with respect to the following aspects: frequency of clusters within genotypes, sizes of clusters, the overlapping number of nucleotides, number of countries involved, period over which outbreaks were reported, transmission modes as reported in the categories foodborne, FHB, person-borne, and UN. On the basis of available information, reported sources of infection in foodborne outbreaks were allocated to the following categories: filter-feeding bivalve shellfish (including oysters and mussels); berries (including raspberries, blueberries, and strawberries); water (including water related to food preparation, irrigation, and contaminating floods, but no shellfish or berries reported); ready-to-eat (including foods like bread, sandwiches, layer cakes, food purchased at a delicatessen, salad, but no shellfish or berries reported as one of the ingredients); and other (including self-served meals, buffet or catering, with multiple food items but none of the previous food classes reported). Outbreak strain sequence clusters that included at least 1 foodborne outbreak were selected for further analysis, and designated “possible foodborne clusters.”

In step 3, we used the APE package of R to stepwise extend all possible foodborne clusters to include sequences with similarities of 99.5%, 99%, 98%, 97%, 96%, and 95%. This was done to determine the cutoff level, i.e., the level of similarity needed to recognize potentially linked outbreaks and thereby to assist in the confirmation of definitely linked outbreaks.

In step 4, p values were calculated to determine association with food for a chosen cutoff level. To do so, the frequencies of the transmission mode for each strain were considered a random draw from the frequencies of this transmission mode in the background population in the database as a null hypothesis, i.e., as random draws from a binomial distribution, and thus the probability of finding such a cluster by chance is low. For example, a cluster of 5 strains that includes 2 foodborne (i.e., 40%) has a probability of 0.02 to be found coincidentally in a total dataset containing 5% foodborne outbreaks. This cluster is then considered to be significantly associated with foodborne transmission. Such calculations were done to determine the following: 1) the association of genotypes with foodborne transmission, i.e., foodborne genotype, with the frequency of foodborne outbreaks in the genotype considered as a random draw from the total dataset; 2) association of clusters with foodborne transmission, i.e., the frequency of the foodborne mode of transmission for the specific cluster considered as a random draw from all strains in the genotype; and 3) association of clusters with a specific food class, i.e., the frequency of the food class for the specific cluster considered as a random sample from all foodborne outbreaks. These calculations were the basis for the transition from possible to probable foodborne clusters.

In step 5, the calculations of step 4 were used to narrow all possible foodborne outbreaks to probable foodborne outbreaks, i.e., those clusters that were more likely to be related to food. The clusters that were significantly and borderline significantly associated with food were selected according to 3 selection criteria: 1) all possible foodborne clusters in foodborne genotypes; and for the non-foodborne genotypes; 2) those possible foodborne clusters that were significantly or borderline significantly associated with foodborne transmission; and 3) those possible foodborne clusters that were significantly or borderline significantly associated with a specific food class. Clusters selected through these criteria were designated probable foodborne clusters; p values were considered significant if p<0.05, and borderline significant if 0.05<p<0.10. In step 6, outbreaks that could be linked and internationally disseminated were selected from probable foodborne clusters if they involved >2 countries.

### Verification of Outcomes

The above selection criteria were applied to the FBVE database to retrospectively identify clusters of outbreaks that may have involved international dissemination of food. To verify the criteria of the approach as described above, the selected clusters were compared with the clusters previously reported to FBVE as linked outbreaks, as a measure of sensitivity of the approach (i.e., ability to detect true clusters).

### Estimate of Proportion of Common-Source Foodborne Outbreaks

The frequency of linked foodborne outbreaks, both at national and international levels, was calculated as a proportion of the total number of reported outbreaks, based on the above analyses. Due to uncertainty of the causal, consequential, or coincidental relationship between outbreaks in molecular clusters, estimates were given for low, most likely, and high values of the number of linked foodborne outbreaks. Low values were calculated as the actual frequency of outbreaks in the specific clusters reported to be foodborne. High numbers were the total number of outbreaks in the specific clusters, i.e., including all reported transmission modes. For the likely values, outbreaks from unknown transmission were extrapolated proportionally to the foodborne outbreaks reported in the specific cluster. Thus, likely values were calculated as follows: for a cluster of *x* outbreaks containing *a* (foodborne), *b* (PTP), and *c* (FHB), and *d* (UN) outbreaks, the high value is *x*, the low value is *a*, and the likely value is



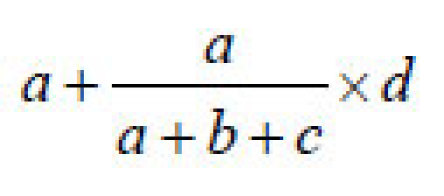



Likely values with range for low and high values were subsequently compared with results of the epidemiologic overviews currently used in outbreak reporting in Europe (24,25).

## Results

### Assignment of Genotypes

Genotyping resulted in clustering of reported outbreak strains into 23 ORF2 genotypes for 1,456 (97%) of 1,504 sequences. For the remaining 48, sequence data provided were insufficient for assignment of a genotype.

### Cluster Analysis

The degree of strain similarity and the proportion of clustering strains (Figure, steps 1 and 2) varied greatly among genotypes (Table A1). A total of 112 clusters of identical (100% similarity) outbreak strains were found, with 938 (64%) of 1,456 reported outbreaks found in clusters. Of these, 38 sequence clusters involving 654 (70%) of 938 outbreaks included at least 1 foodborne outbreak. These were designated possible foodborne clusters, i.e., possibly representing linked foodborne outbreaks (Figure, step 2).

When the cutoff for strain similarity was lowered step-wise in R (Figure, step 3), logically, the number of distinctive clusters decreased, whereas the size of each cluster increased. The similarity cutoff differed between genotypes. Six genotypes (I.1, I.4, I.5, II.1, II.5, and II.8) yielded a cluster of strains that remained distinct regardless of the cutoff used. For the other genotypes, lowering the cutoff to similarity levels of 99.5% or 99% showed a sharp drop in the number of distinct clusters, i.e., fewer clusters; as a consequence, clusters increased in size. For 7/14 genotypes the number of such clusters dropped to 50% at cutoff value of 99.5%. At 99%, this was the case for 10/14 genotypes (data not shown). Because we aimed to provide a conservative estimate for linked outbreaks for all genotypes, 100% similarity was chosen as the cutoff for further analysis steps.

Probable foodborne clusters of outbreaks were selected from 38 possible foodborne clusters based on 3 criteria for statistical association with food (Figure, step 4; Table A2): 1) twenty-two clusters in 8 genotypes (I.1, I.4, I.5, I.6, II.1, II.2, II.6, II.7, II.8) significantly or borderline significantly more often contained foodborne outbreaks, compared with the total dataset; 2) five additional clusters showed nonfoodborne genotypes for which the specific transmission mode foodborne was more frequently reported than in the genotype; and 3) two additional clusters were associated with a food class, compared with the frequency of these food classes reported for all foodborne outbreaks. Fourteen of these 29 probable foodborne clusters involved >1 country and were therefore labeled as probable internationally disseminated foodborne outbreaks (Figure, step 6).

### Validation of Criteria

In the FBVE dataset of 1,456 capsid sequences, 36 outbreaks had previously been identified as linked outbreaks in 10 clusters, based on standard epidemiologic investigation (24). In contrast, in the present study, 29 clusters of interest involving 122 likely linked outbreaks (range 51–166) were retrospectively identified (Table A2). Of the 10 previously reported FBVE outbreak clusters, 8 were identified by using the approach described in this paper. These 8 clusters involved 32 likely linked outbreaks (range 18–69) and included 2 international and 3 national clusters, plus 3 clusters reported as national but containing sequences identical to those from outbreak strains reported elsewhere. The 2 FBVE clusters that were missed by our analysis involved 3 outbreaks with 3 different genotypes involved, and 3 outbreaks for which 2 different food classes were reported (ready-to-eat and other). Both food classes ended up nonsignificant for this cluster in step 5 (Figure) of the analysis.

### International Clusters Potentially Linked through a Common Source

Previously, 36 of 1,456 (2.5%) outbreaks reported through the FBVE network had been linked to a common source, of which 6 (0.4%) involved events in multiple countries. Our use of the stepwise criteria described here resulted in a significant increase to 122 of 1,456 (8.4%, range low-high: 51–166) potential common-source outbreaks, of which 97 (6.7%, range 29–130) involved events in >1 country (data not shown).

## Discussion

Our analysis suggests that 7% (range 2%–9%) of norovirus outbreaks reported through the FBVE network are likely to be international outbreaks with a common source. Our estimate is at least 5-fold higher than the 0.4% recognized through routine investigations. We showed that the proportion of linked foodborne outbreaks can be estimated with a sensitivity of 80% by using step-wise selection criteria combining molecular and epidemiologic information and derived from a large background dataset. The computerized linking of epidemiologic data to aligned sequences in R project for statistical computing considerably reduced the time needed for analysis and was an essential prerequisite of this novel approach. As sequencing becomes less expensive and public health databases expand, the utility of our approach for public health decision-making will increase (34).

Several research groups have made efforts to estimate the public health effects of norovirus and foodborne disease, finding that viral illness varies between 1/780 UK inhabitants and 1/33 US inhabitants (1,35–37). For Europe, we previously estimated that 21% of all norovirus outbreaks were caused by food (38), but that report did not consider potential (international) links between outbreaks. In our current study, we found that 2%–9% of all reported outbreaks may be linked to a common source with international distribution. Because this study was done retrospectively, we could not collect additional data to verify suspected clusters. To prove this with certainty, the analysis should be done in real time and involve more in-depth outbreak investigations to establish a risk food with epidemiologic approaches and possibly food testing. However, we do see this as a novel approach to provide the basis for estimates of the prevalence and public health consequences of foodborne disease. Past studies have used gross extrapolations of data estimating the proportion of reported noroviral disease that can be attributed to food, but have not included the effect of outbreaks. We suggest a basis for such estimates, and especially the proportion attributable to foodborne transmission.

Our approach most likely provides a conservative estimate, because it relies on identical sequence clusters and does not include outbreaks caused by strains that are phylogenetically closely related. Given the mutation rate of genotype II.4 noroviruses (39), closely related strains could well represent linked outbreaks. This mutation rate, as well as the similarity cutoff, may be genogroup or genotype specific (Table A1). When a single similarity cutoff for all genotypes is used as a selection criterion, any mutation counts equally. Nevertheless, a particular mutation may indicate that strains share a common ancestor, and a mutation in a particular genotype may indicate either a longer or shorter genetic distance. Therefore, phylogenetic analysis is needed to identify additional linked outbreaks involving closely related strains.

A shortcoming of our methods is that we would miss common source events that involve >1 strain, as has been described in some examples that involved sewage-contaminated shellfish (6,19,40). Nevertheless, we detected 3 of 4 linked outbreaks involving multiple genotypes, which indicates that such outbreaks are likely to show other characteristics that can be captured by our criteria.

A prerequisite for our approach is the availability of combined epidemiologic and laboratory data. National surveillance systems differ in their potential for matching these data, in the intensity of surveillance, and in the attention given to foodborne outbreaks. With no special focus, foodborne outbreaks are likely to be underreported, as recognition will be complicated by rapid emergence of PTP transmission (10). Moreover, many persons are involved in data entry, which may have had consequences for data quality because of human error (24). Unevenness of data quality may hamper international comparisons to detect foodborne outbreaks (25). For instance, France and Denmark have been reporting primarily foodborne outbreaks, whereas other countries include a wider range of transmission modes. An added value of our approach is reflected in its finding of foodborne outbreaks in France and Denmark and in other countries as well, e.g., the United Kingdom and Germany, which are less focused on identification of foodborne outbreaks. Despite the fact that underreporting of foodborne outbreaks in our dataset was likely, our criteria may thus provide insight into the number of foodborne outbreaks occurring in countries whose surveillance systems may miss such outbreaks.

In conclusion, combined epidemiologic and molecular analysis can recognize internationally disseminated outbreaks that may share a common foodborne source. Step-wise selection criteria can be derived from an extensive background dataset and used to retrospectively estimate the proportion of international outbreaks that share a foodborne source. Prospective use of the criteria needs to be validated through real-time data sharing and timely follow-up of outbreak clusters. Our findings nevertheless show that current surveillance has a critical gap, which can be bridged through systematic analysis of combined molecular and epidemiologic data.

On behalf of the Foodborne Viruses in Europe Network, the following persons contributed to the work described in this paper: the Netherlands: E. Duizer (RIVM); United Kingdom: D. Brown, B. Adak, J. Gray, J. Harris, M. Iturriza (Health Protection Agency); Finland: K.-H. von Bonsdorff, L. Maunula (University of Helsinki), and M. Kuusi (National Public Health Institute); Denmark: B. Böttiger, K. Mølbak, C. Johnsen (Statens Serum Institut); Sweden: K.-O. Hedlund, Y. Andersson, M. Thorhagen, M. Lysén, M. Hjertqvist (Swedish Institute for Infectious Disease Control); France: P. Pothier, E. Kohli, K. Balay, J. Kaplon, G. Belliot (University of Dijon), and S. Le Guyader (Institut Françai pour la Recherche et l’Exploitation de la Mer); Spain: A Bosch, A.Dominguez (University of Barcelona), J. Buesa (University of Valenica), A. Sanchez Fauquier and G. Hernández-Pezzi (Instituto de Salud Carlos III); Hungary: G. Szücs, G. Reuter (State Public Health Service), and K. Krisztalovics (National Center for Epidemiology); Slovenia: M. Poljsak-Prijatelj, D. Barlic-Maganja (University of Ljubljana) and A. Hocevar Grom (Institute of Public Health of the Republic of Slovenia); Italy: F. Ruggeri, and I. Di Bartolo (Instituto Superiore di Sanita); Germany: E. Schreier, K. Stark, J. Koch, M. Höhne (Robert Koch Institute); Ireland: M. Lynch (Mater Misericordiae Hospital); B. Foley, P. McKeown (Health Protection Surveillance Center); S. Coughlan (National Virus Reference Laboratory); Norway: K. Vainio, K. Nygard, and G. Kapperud (Norwegian Institute of Public Health)

## Appendix

**Table A1:** Summary of aggregated data of clustered outbreak strains, sequences, and properties of the outbreaks within such clusters for each of the genotypes, 1999–2008.*

GT	Total no. strains	No. (%) strains in clusters	No. (%) strains not in clusters	Minimum similarity within GT, %	Total no. clusters	Cluster size, range†	Cluster size, median†	No. (%) FB outbreak strains	No. sign possible FB clusters	No. (%) FHB outbreak strains	No. sign FHB clusters	No. (%) PB outbreak strains	No. sign PB clusters	No. (%) UN outbreak strains	No. sign UN clusters
I.1	24	19 (79)	5 (21)	91.2	4	2–7	5	8 (33)‡	1¶	0	0	2 (8)	0	14 (58)	0
I.2	34	20 (59)	14 (41)	97.5	5	2–6	5	5 (15)	1¶	0	0	0	0	29 (85)§	0
I.3	43	16 (37)	27 (63)	78.3	5	2–6	4	6 (14)	1¶	0	0	2 (5)	0	35 (81)	0
I.4	40	23 (58)	17 (42)	94.8	8	2–5	2	8 (20)‡	1¶	2 (5)‡	1¶	0	0	30 (75)	0
I.5	6	5 (83)	1 (17)	93.1	2	2–3	2.5	2 (33)§	0	0	0	0	0	4 (67)	0
I.6	20	13 (65)	7 (35)	88.7	4	2–5	3	4 (20)§	1#	1 (5)§	0	2 (10)	0	13 (65)	0
II.1**	28	10 (36)	18 (64)	90.9	4	2–3	2.5	6 (21)‡	0	0	0	12 (43)‡	0	10 (36)	0
II.2	30	9 (29)	21 (71)	82.4	4	2–3	2	11 (37)‡	0	0	0	3 (10)	0	16 (53)	0
II.3**	136	70 (52)	66 (48)	90.5	14	2–15	3	15 (11)	0	0	0	35 (26)‡	4¶#	86 (63)	0
II.4**	950	691 (73)	254 (27)	82.4	47	2–361	3	41 (4)	3¶#	2††	0	178 (19)§	3¶#	729 (77)‡	4¶#
II.5	3	2 (66)	1 (33)	97.0	1	2	2	1 (33)	0	0	0	0 (0)	0	2 (67)	0
II.6**	54	30 (56)	24 (44)	92.0	8	2–15	2	11 (20)‡	1¶	1 (2)	0	6 (11)	1¶	36 (67)	0
II.7	57	39 (68)	18 (32)	96.2	4	2–28	3	13 (23)‡	1#	0	0	6 (11)	1¶	38 (67)	0
II.8	4	2 (50)	2 (50)	96.0	1	2	2	2 (50)‡	0	0	0	0 (0)	0	2 (50)	0
II.10	3	0	3 (100)	97.0	0	NA	NA	0	0	0	0	2 (67)§	0	1 (33)	0
II.11	1	0	1 (100)	NA	0	NA	NA	0	0	0	0	0	0	1 (100)	0
II.12	4	0	4 (100)	93.9	0	NA	NA	0	0	0	0	0	0	4 (100)	0
II.13	3	0	3 (100)	96.6	0	NA	NA	1 (33)	0	0	0	0	0	2 (67)	0
II.14	3	0	3 (100)	99.5	0	NA	NA	1 (33)	0	0	0	0	0	2 (67)	0
II.16	4	0	4 (100)	97.5	0	NA	NA	0	0	0	0	1 (25)	0	3 (75)	0
II.17	1	0	1 (100)	NA	0	NA	NA	0	0	0	0	0	0	1 (100)	0
II.NA1	6	2 (50)	4 (50)	94.2	1	2	2	0	0	0	0	2 (50)	0	2 (50)	0
II.NA3	1	0	1 (100)	NA	0	NA	NA	0	0	0	0	0	0	1 (100)	0
Overall	1456	938 (64)	518 (36)		112	2–364		136 (9)	10	6††	1	252 (17)	9	1062 (73)	4

*GT, genotype; FB, foodborne; FHB, food-handler borne; PB, person-borne; UN, unknown; NA, not applicable. †Size of a strain cluster is the number of outbreak-representative strains within a molecular cluster of outbreaks, i.e., 2–7 outbreaks (not 2–7 patients per outbreak) ‡Genotypes with significantly higher proportion (p<0.05) of outbreaks with this transmission mode, when compared to the proportion in the total sequence population. §Genotypes with borderline significantly higher proportion (0.05<p <0.10) of outbreaks with this transmission mode, when compared to the proportion in the total sequence population. ¶Clusters with significantly higher proportion (p < 0.05) of outbreaks with this transmission mode, when compared to the proportion within the genotype. #Clusters with borderline significantly higher proportion (0.05<p<0.10) of outbreaks with this transmission mode, when compared to the proportion within the genotype. **Genotypes II.1, II.3, II.4 and II.6 were aligned separately for different genomic regions. ††Percentage values <0.5.

**Table A2:** Characteristics of 29 strain sequence clusters with identical capsid sequences that may share a common exposure through food, 1999–2008*

Cluster	No. strains (% total in GT)	Minimum no. NT overlap	Transmission mode based on reports† (p value)	Source category	Time span	Countries involved	Reported to FBVE as linked outbreak
Selection criterion 1: identical clusters within FB genotypes including at least 1 FB outbreak, n = 22							
1 (I.1)	7 (29)	204	UN? (0.38)	Shellfish	2004 Sep–Dec and Feb 2006	FR, HU, GB	Yes, in FR
2 (I.1)	4 (17)	204	FB (0.01)	Shellfish	2000 Apr–Nov and 2001	FR, NL	Yes, in FR
3 (I.1)	2 (8)	206	FB? (0.11)	Unknown	2000	FR	No
**4 (I.1)**	**6 (25)**	**221**	**UN? (0.20)**	**Unknown**	**2000 Dec–2005 Dec and 2006**	**FR, GB, SE**	**No**
**5 (I.4)**	**5 (13)**	**204**	**FB? (0.45)**	**Water, berries**	**2000 and 2002 Feb–2006 Aug**	**NL, FR, SE**	**No**
6 (I.4)	2 (5)	204	FB (0.04)	Shellfish	2000 and 2001	NL	No
7 (I.4)	2 (5)	215	FHB (0.00)	RTE	Mar 2006	DK	Yes
**8 (I.5)**	**3 (50)**	**204**	**FB? (0.25)**	**Shellfish, delicatessen**	**2004 Nov–2006 Feb**	**IE, FR**	**No**
**9 (I.6)**	**5 (25)**	**204**	**FB (0.07)**	**Self-served meal, shellfish**	**2002 Dec–2006 Jul**	**HU, NL, SE**	**No**
10 (I.6)	3 (13)	291	FHB? (0.14)	Self-served meal	2002 Sep and 2004	SE	No
11 (II.1)	3 (11)	272	FB? (0.11)	Water	2000 Sep–2001 Jan	FR	No
12 (II.1)	3 (11)	268	FB?(0.29)	Unknown	2001 Jan–Mar	NL	No
13 (II.2)	2 (6)	255	FB? (0.14)	Shellfish	2006 Dec and 2007 Mar	FR	No
**14 (II.2)**	**2 (6)**	**235**	**FB? (0.60)**	**Shellfish**	**2005 Jan and 2008 Feb**	**FR, GB**	**No**
15 (II.2)	2 (6)	338	FB? (0.60)	Water	2002 Oct and 2003 Mar	SE	No
16 (II.6)	2 (4)	255	FB (0.04)	Paella	2007 Sep	FR	Yes
17 (II.6)	15 (28)	45	PTP? (0.22)	Berries, RTE	2004 Jan–2006 Dec	HU, SE, DK, GB, FR, NL, IT	Yes, multiple countries and genotypes
**18 (II.6)**	**2 (4)**	**196**	**FB? (0.36)**	**Self-served meal**	**2006 Jan–May**	**GB, NL**	**No**
**19 (II.7)**	**28 (49)**	**94**	**UN?(0.55)**	**Self-served meal, RTE**	**2003 Jun–2006 Apr**	**GB, DK, NL, FR, SE**	**No**
20 (II.7)	2 (4)	337	FB (0.05)	RTE	2004 Feb	SE	No
21 (II.7)	5 (9)	249	PTP (0.01)	RTE	2005 Dec–2006 Mar	SE	No
22 (II.8)	2 (50)	338	FB? (0.25)	berries	2006 Jun and Aug	SE, PL	Yes, multiple genotypes, link abroad
Selection criterion 2: identical clusters significantly associated with FB transmission, n = 5							
23 (I.2)	2 (6)	206	FB (0.02)	Unknown	2000 Apr	FR	No
24 (I.3)	2 (5)	206	FB (0.02)	Shellfish	2000 Apr	FR	No
25 (II.4)	35 (4)	140	FB (0.05)	RTE, shellfish, berries	2006 Jan–2006 Nov	FR, DK, SL, IE, HU, SE, IT, NL	Yes, local link hospitals
26 (II.4)	2 (0)	238	FB (0.08)	Unknown	2008 Feb	FR	Yes
27 (II.4)	2 (0)	249	FB (0.08)	Unknown	2000 Nov and 2001 Dec	NL	No
Selection criterion 3: identical clusters statistically associated with a specific food class (shellfish, berries, water, other), n = 2							
**28 (I.3)**	**4 (9)**	**234**	**FB? (0.45)**	**Water, berries**	**2000 and 2002 Feb–2006 Aug**	**FR, DE, SE**	**No**
**29 (II.4)**	**12 (1)**	**227**	**UN? (0.20)**	**Shellfish**	**2007 Oct–2009 Nov**	**FR, IE**	**No**

*Outbreak clusters were selected based on one of 3 criteria: 2a) genotype preferentially found in food-borne outbreaks; 2b) food-borne mode of transmission more commonly reported for cluster and 2c) specific food item more commonly reported in given cluster of outbreaks. Outbreak clusters in **boldface** were international outbreaks newly identified through the analysis in this study, other international outbreak clusters had already been reported as (suspected) common source events. FBVE, Food-Borne Viruses in Europe; GT, genotype; UN, unknown; FR, France; HU, Hungary; GB, Great Britain; FB, foodborne; NL, Netherlands; SE, Sweden; FHB, foodhandler-borne; RTE, ready-to-eat; DK, Denmark; IE, Ireland; PTP, person-to-person; IT, Italy; PL, Poland. †Frequency of transmission modes as reported for the single outbreaks within the specific cluster were considered a random draw from the frequencies of this transmission mode from the background population in the Food-borne Viruses in Europe database, i.e., as random draws from a binomial distribution. p values <0.10 were considered significant or borderline significant. p values >0.10 were considered nonsignificant. However, for clusters with none of the transmission modes ending up with a significant p value, the transmission mode with the smallest p value was chosen and presented with a question mark (?).
